# Relationship between serum NDRG3 and papillary thyroid carcinoma

**DOI:** 10.3389/fendo.2022.1091462

**Published:** 2022-12-20

**Authors:** Jiahao Wang, Jun Wang, Jinxing Quan, Juxiang Liu, Limin Tian, Changhong Dong

**Affiliations:** ^1^ The First Clinical College of Gansu University of Chinese Medicine, Lanzhou, Gansu, China; ^2^ Department of Thyroid and Breast Surgery, Gansu Cancer Hospital, Lanzhou, Gansu, China; ^3^ Department of Endocrinology in Gansu Provincial People’s Hospital and The First Clinical College of Gansu University of Chinese Medicine, Lanzhou, Gansu, China; ^4^ Radiotherapy Department of Gansu Maternal and Child Health Hospital, Lanzhou, Gansu, China

**Keywords:** benign thyroid nodules, papillary thyroid carcinoma, NDRG3, differential diagnosis value, molecular marker

## Abstract

**Background:**

In recent years, papillary thyroid carcinoma is considered to be one of the fastest increaseing cancer. NDRG family member 3 (NDRG3) has been proposed as a molecular marker of tumor, and is expected to be used in clinic.

**Methods:**

Enzyme-linked immunosorbent assay was used to detect the serum NDRG3 expression in 81 papillary thyroid carcinoma cases, 75 benign thyroid nodules cases and 77 healthy control cases, respectively. Electrochemiluminescence method was applied to measure the levels of triiodothyronine, tetraiodothyronine, thyrotropin, thyroglobulin antibody and thyroid peroxidase antibody. Immunohistochemical staining was used to detect the expression of NDRG3 in papillary thyroid carcinoma, benign thyroid nodules and normal tissues adjacent to cancer.

**Results:**

The expression of serum triiodothyronine, tetraiodothyronine, thyrotropin, thyroglobulin antibody and thyroid peroxidase antibody and NDRG3 were significantly different among benign thyroid nodules, papillary thyroid carcinoma cases and healthy control groups (*P <*0.001). Only the expression of serum NDRG3 was significantly different between benign thyroid nodules and papillary thyroid carcinoma groups (*P <*0.001). Immunohistochemistry showed that NDRG3 was expressed in all three groups, the lowest in papillary thyroid carcinoma, the second in benign thyroid nodules, and the highest in normal tissues adjacent to cancer. Logistic regression analysis showed that serum NDRG3 was an independent protective factor for papillary thyroid carcinoma (OR =0.964, 95%CI =0.953 to 0.974, *P <*0.001). The ROC curve of non-papillary thyroid carcinoma diagnosed by serum NDRG3 showed the optimal cut-off value of 481.38 pg/ml, sensitivity of 72.4%, specificity of 90.1%, and the maximum area under the curve (AUC =0.902, 95%CI =0.863 to 0.940, *P <*0.001). The ROC curve of benign thyroid nodules diagnosed by serum NDRG3 showed the optimal critical value of 459.28 pg/ml, sensitivity of 81.3%, and specificity of 74.1% (AUC =0.863, 95%CI =0.808 to 0.919, *P <*0.001). The expression level of serum NDRG3 was significantly correlated with extrathyroid extensionand (*P =*0.007) and lymphatic metastasis of papillary thyroid carcinoma (*P* =0.019).

**Conclusions:**

The decrease of NDRG3 expression can not only differential diagnosis benign thyroid nodules and papillary thyroid carcinoma, but also serve as a molecular marker for the diagnosis of papillary thyroid carcinoma.

## Introduction

Thyroid nodule is the most common thyroid disease, with an increasing global morbidity in recent years, which is rising with the progress of detection methods (1, 2). Ultrasound is the optimal imaging method for thyroid examination (3). The prevalence of ultrasound has increased the morbidity of thyroid nodules at home and abroad, but may lead to overdiagnosis and unnecessary intervention (4, 5). Since more than 90% of thyroid nodules are asymptomatic benign lesions, only 10% of thyroid nodules are susceptible to thyroid cancer (4, 6). Despite the accuracy and economic applicability, there still exists 2-16% interpreted uncertainty of fine needle aspiration cytology to distinguish benign from malignant thyroid nodules (7). In the past 20 years, the incidental thyroid nodules detected by ultrasound have led to a rapid increase in the morbidity of low-risk papillary thyroid cancer (8). It is estimated that, thyroid cancer will become the four most common malignancies by 2030 (9). Although the morbidity of thyroid cancer has tripled in the past three decades (10, 11), the mortality rate remains relatively stable (12, 13). Papillary thyroid carcinoma (PTC), accounting for 84% of thyroid cancers, is the most common histopathological type of thyroid cancer. Although PTC patients can achieve a favourable prognosis, the 5-year survival rate generally exceeds 97%, and the 10-year survival rate exceeds 90% (14). In addition, there are still a significant number of patients with invasive metastasis, recurrence and iodine-131 resistance, resulting in adverse outcomes. For instance, studies have found that approximately 10-15% of patients may progress to potentially fatal recurrent diseases (15, 16). Accurate preoperative clinical diagnosis has proved difficult due to the lack of specific diagnostic tests for PTC. Therefore, there is an urgent need for a convenient, non-invasive and specific diagnostic method to distinguish PTC from benign thyroid nodules (BTN).

Molecular markers are expected to be used for early detection or screening of thyroid cancer, differentiation of benign and malignant diseases, histological identification, staging and treatment response, diagnosis and prognosis of recurrence (17). The American thyroid Association and the European thyroid Association have published guidelines recommending the detection of molecular markers for uncertain nodules (18). MYC is a human proto-oncogene, including C-MYC, N-MYC and L-MYC, involved in the occurrence, development, invasion and metastasis of tumors. Abnormal expression of MYC in PTC has been reported in a slew of literatures (19). The NDRG family is a downstream regulator of MYC. The AceView database of NCBI showed that, NDRG family members have a NDR-α/β hydrolase folding region and several functional sites, such as phosphorylation sites, acetylation sites, ubiquitin sites, etc. The members of the NDRG family are intracellular proteins, which are composed of 340-394 amino acid residues with amino acid homology of 53-65%. The NDRG family is generally divided into two subfamilies, one composed of NDRG1 and NDRG3 with a homology of 67%, and the other composed of NDRG2 and NDRG4 with a homology of 58% (20). NDRG3 is a downstream regulatory factor of MYC, with a total length of 2588 base pairs. It is mainly expressed in ovary, prostate, testis, brain, spinal cord, thymus, heart and kidney (21). NDRG3 is located on chromosome 20q11.21-11.23 and encodes at least two subtypes, 375 and 363 amino acids, respectively, with an apparent molecular weight of 41 and 40 kDa, respectively (22). However, the relationship between NDRG3 and PTC has not been reported.

Therefore, this study aimed to investigate the differential diagnostic value of NDRG3 in patients with BTN and PTC by detecting the serum and tissues expression level of NDRG3 in normal individuals, BTN and PTC patients, and analyze the relationship between the serum expression level of NDRG3 and clinicopathology of PTC.

## Materials and methods

### Study subjects

From June 2019 to June 2022, patients with thyroid nodules underwent an operation in the Department of Head and Neck Surgery, Gansu Cancer Hospital and Gansu people’s Hospital were divided into BTN group (n =75, 17 males and 58 females), and PTC group (n =81, 20 males and 61 females) according to pathological results. These patients did not have any other diseases before diagnosis and had not received any other treatment before the operation. In the same period, 77 healthy volunteers who underwent physical examination in the Physical Examination Center of Gansu Provincial People’s Hospital were selected as the healthy control group (HC). The HC group received thyroid function, ultrasound and other examinations to exclude any disease, including 21 men and 56 women. All specimens were obtained with written informed consent. The study was designed to comply with the ethical standards set out in the 1975 Helsinki Declaration and was approved by the Institution Review Board of the Hospital (IRB No. 2022-224).

### Data collection

Medical data of gender and age were recorded in detail for all subjects. Fasting blood was collected from all subjects, and thyroid function triiodothyronine (T3), tetraiodothyronine (T4), thyrotropin (TSH), thyroglobulin antibody (TGAB) and thyroid peroxidase antibody (TPOAb) was detected by chemiluminescence. 5 ml of total blood from the cubital vein was collected 2-3 days before operation. The specimens were placed in a vacuum blood collection jar without anticoagulant, refrigeratea at 4°C and centrifuged at 3000 r/min for 72 hours. After 10-15min, the supernatant was taken, placed in a centrifuge tube and stored in a refrigerator at -80°C. The postoperative pathological tissue was collected, fixed with 4% formaldehyde, washed, dehydrated, transparent, impregnated and embedded, and paraffin sections were prepared.

### Main reagents and sources

Serum NDRG3 concentration was detected by Enzyme-linked immunosorbent assay (ELISA) kit, purchased from Shanghai FANKEL Industrial Co., Ltd. The antibody of NDRG3 was purchased from Affinity Biosciences company.

### Experimental method

The main steps were as follows: the prepared standards and specimens were added to the microwell plate and shaken at 37°C for 30 min. After washing the microplate, 50 μL of secondary antibody was added and placed at room temperature for 30 min. After washing, the substrate was added, the color was shaded at 37°C for 10 minutes, and then the termination solution was added. Absorbance (OD) was measured with a microplate reader at a wavelength of 450 nm, and the concentration of human NDRG3 in the specimens was calculated with a standard curve. Sections of 6 cases of PTC, paracancerous normal tissues and BTN were randomly selected and the immunohistochemical experiment was carried out by Shaanxi Yike Biotechnology Co., Ltd. The results showed that the nucleus stained with hematoxylin was blue and the positive expression of diaminodiamine(DAB) was brown.

### Statistical methods

SPSS25.0 (IBM, Armonk, NY, USA) was used for data analysis. Non-normal distribution data were described by the median (interquartile range [IQR]: 25–75 percentiles), difference analysis was carried out by Kruskal-Wallis test, and pairwise comparison was tested by Mann-WhitneyU test. Counting data were described in the form of frequency (percentage), and chi-square analysis was used for difference analysis. The immunohistochemical images were analyzed by Image-pro plus, and the data obtained were processed by one-way ANOVA. *P <*0.05 was considered statistically significant.

## Results

### Comparison of baseline characteristics and experimental indexes

As shown in **Table 1**, the index levels of 81 PTC patients, 75 BTN patients, and 77 healthy individuals were compared. The difference analysis of age and gender showed that no significant difference in this variable among BTN, PTC and HC groups (*P >*0.05). The difference analysis of T3, T4, TSH, TGAb and TPOAb showed that there were significant differences in this variable among BTN, PTC and HC (all *P <*0.001), between HC and PTC (all *P <*0.001), and between HC and BTN (all *P <*0.05). However, there was no significant difference between BTN and PTC (all *P >*0.05). The difference analysis of NDRG3 showed significant difference in this variable among BTN, PTC and HC, HC and PTC, BTN and PTC, and HC and BTN (all *P <*0.001).

**Table 1:** Baseline Characteristics and comparison of experimental indexes.

Characteristics	PTC (n=81)	BTN (n=75)	HC (n=77)	*P*-Value
Gender				
Female(%)	61 (75.3%))	58 (77.3%)	56 (72.7%)	0.805
male(%)	20 (24.7%)	17 (22.7%)	21 (27.3%)	
Age(years)	45 (37, 54)	47 (38, 55)	44 (35, 48)	0.073
T3(nmol/L)	2.04 (1.92, 2.25)^*^	2.03 (1.83, 2.31)^*^	1.51 (1.37, 1.69)	<0.001
T4(nmol/L)	117.20 (101.80, 131.35)^*^	113.30 (98.83, 122.80^)*^	99.35 (88.76, 110.37)	<0.001
TSH(uIU/L)	2.95 (1.77, 4.33)^*^	2.52 (1.59, 3.90)^*^	1.99 (1.28, 2.83)	<0.001
TGAb(IU/ml)	22.00 (18.18, 38.84)^*^	19.55 (14.84, 29.77)^*^	1.47 (0.84, 2.37)	<0.001
TPOAb(IU/ml)	3.21 (2.24, 13.22)^*^	3.57 (1.99, 5.18)^*^	0.50 (0.30, 1.15)	<0.001
NDRG3(pg/ml)	420.89 (375.57, 460.00)^*#^	490.45 (461.28, 517.60)^*^	532.05 (499.86, 583.42)	<0.001

PTC, Papillary thyroid carcinoma; BTN, Benign thyroid nodules; HC, Healthy controls; T3, Triiodothyronine; T4, tetraiodothyronine; TSH, thyrotropin; TGAb, thyroglobulin antibody; TPOAb, thyroidperoxidase antibody; ^*^represents a statistically significant difference compared with the HC, and ^#^represents a statistically significant difference compared with the BTN.

### Immunohistochemistry

**Figure 1:**
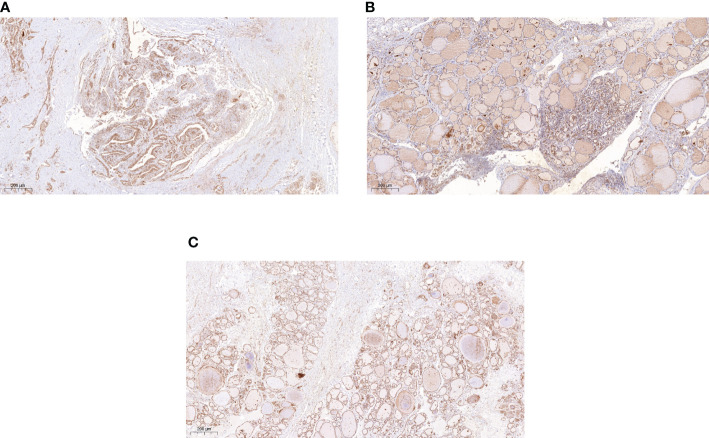
The expression of NDRG3 in PTC, paracancerous normal tissues and BTN (200X) **(A)** Positive expression of NDRG3 in PTC tissues; **(B)** Positive expression of NDRG3 in normal tissues adjacent to cancer; **(C)** Positive expression of NDRG3 in BTN tissues. The nucleus and cytoplasm are stained.

Thyroid follicular epithelial cells and cancer cells showed positive expression of NDRG3, and the nucleus and cytoplasm were stained brown (**Figure 1**). There was significant difference in NDRG3 between benign nodule group and adjacent normal tissue (*P <*0.001), between PTC group and adjacent normal tissue (*P* < 0.001), and between nodule group and PTC group (*P <*0.01).

### Relationship between each experimental index and PTC

Spearman correlation analysis of PTC and other factors showed that serum levels of T3, T4, TSH, TGAb, and TPOAb were positively correlated with PTC (r =0.562, *P* =0.002; r =0.360, *P <*0.001; r =0.254, *P <*0.001; r =0.711, *P <*0.001; r =0.605, *P <*0.001; r =0.489, *P <*0.001). However, NDRG3 was significantly negative correlated with PTC (r =-0.700, *P <*0.001). After adjusting for T3, T4, TSH, TGAb, TPOAb and other factors, logical regression analysis showed that elevated serum NDRG3 expression was an independent protective factor for PTC (OR =0.964, 95%CI =0.953 to 0.974, *P <*0.001) (**Table 2**).

**Table 2:** Binary logistic regression Analysis of PTC and various factors.

Variables	B	S.E.	Wald	Sig.	Exp (B)	95% C.I. for EXP (B)	
						Lower	Upper
T3	0.990	0.061	2.663	0.103	1.104	0.980	1.243
T4	0.006	0.010	0.336	0.562	1.006	0.986	1.027
TSH	0.135	0.107	1.588	0.208	1.145	0.928	1.413
TGAb	0.003	0.003	0.939	0.333	1.003	0.997	1.008
TPOAb	0.028	0.015	3.417	0.065	1.029	0.998	1.060
NDRG3	-0.037	0.006	45.024	0.000	0.964	0.953	0.974

T3, Triiodothyronine; T4, tetraiodothyronine; TSH, thyrotropin; TGAb, thyroglobulin antibody; TPOAb, thyroidperoxidase antibody.

### Diagnostic efficacy of serum NDRG3 between benign and malignant nodules

**Figure 2:**
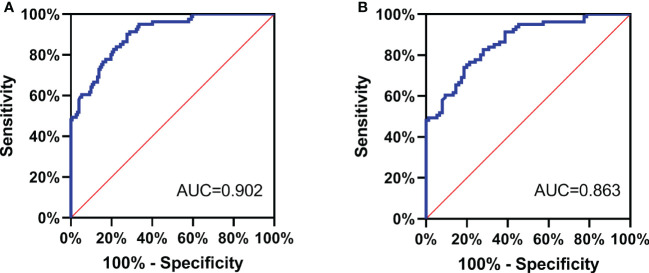
**(A)** All the subjects were divided into PTC group and non-PTC group. The serum NDRG3 was used as a test variable, and the group non-PTC as the state variable to plot the ROC curve; **(B)** In the PTC and BTN groups, serum NDRG3 was used as the test variable and BTN as the state variable, so as to plot the ROC curve.

All the subjects were divided into PTC group and non-PTC group. The serum NDRG3 was used as a test variable, and the group non-PTC as the state variable to plot the ROC curve (**Figure 2A**). The optimal cut-off value of NDRG3 for the diagnosis of non-PTC group was 481.38 pg/ml, the sensitivity was 72.4%, the specificity was 90.1%, and the maximum area under the curve (AUC =0.902, 95%CI =0.863 to 0.940, *P <*0.001).

In the PTC and BTN groups, serum NDRG3 was used as the test variable and BTN as the state variable, so as to plot the ROC curve (**Figure 2B**). The optimal cut-off value of NDRG3 for BTN diagnosis was 459.28pg/ml, with a sensitivity of 81.3%, a specificity of 74.1%, and a maximum area under the curve (AUC =0.863, 95%CI =0.808 to 0.919, *P <*0.001).

### Clinicopathological relationship between NDRG3 and PTC

As shown in **Table 3**, among the 81 PTC patients, the serum NDRG3 levels of PTC patients with different clinicopathological features were compared: ①Gender: 20 males and 61 females; ②Age: 63 patients aged <55 years and 18 patients aged ≥55 years; ③Degree of tumor invasion: 64 cases of PTC had unilateral invasion and 17 cases had bilateral invasion; ④Tumor length: 60 patients ≤10mm and 21 patients >10mm; ⑤Extrathyroidal extension: present in 28 patients and absent in 53 patients; ⑥Lymphatic metastasis: present in 15 patients and absent in 66 patients. In terms of TNM staging, there were 62 cases with stage I to II and 19 cases with stage III to IV, with the basis of the AJCC 8th edition criteria for diagnosis and staging of thyroid cancer. The level of serum NDRG3 in patients with lymph node metastasis and extrathyroidal extension PTC was significantly lower than that in patients without lymph node metastasis (*P =*0.019) and extrathyroidal extension (*P =*0.007). There was no significant difference in serum NDRG3 level in PTC patients with different gender, age, length, TNM stage and tumor invasion site.

**Table 3:** Relationship between NDRG3 and clinical parameters of patients with PTC.

Characteristics	Case	NDRG3	Z-value	*P*-value
Gender				0.706
Male	20	436.943 (365.832, 469.554)	-0.378	
Female	61	417.366 (379.069, 458.137)		
Age(year)				0.478
<55	63	414.179 (376.072, 458.993)	-0.710	
≥55	18	431.234 (361.265, 472.580)		
Location of involvement				0.339
Unilateral	64	421.599 (380.211, 460.498)	-0.957	
Bilateral	17	417.366 (355.592, 450.501)		
Tumor size(mm)				0.242
≤10mm	60	427.808 (376.286, 461.847)	-1.169	
>10mm	21	397.052 (368.294, 440.154)		
Extrathyroidal extension				0.007
yes	28	393.413 (346.029, 433.696)	-2.711	
no	53	433.731 (394.554, 462.701)		
Lymph node metastasis				0.019
yes	15	379.926 (347.099, 432.304)	-2.347	
no	66	427.808 (392.307, 462.346)		
TNM stages				0.061
I-II	62	429.164 (380.354, 462.346)	-1.873	
III-IV	19	397.052 (347.099, 434.159)		

Values are presented as median (interquartile range).

## Discussion

There is an ever-growing global morbidity of thyroid nodules with each passing year. Surgery remains the foremost choice of treatment. The high diagnostic rate of uncertain nodules imposes an unbearable economic burden on individuals and society, and significantly reduces the quality of life. Therefore, it is a clinical challenge to perform a surgery on patients with a risk of cancer, and unnecessary surgery should be avoided. It has been pointed out that, for patients with uncertain nodules, the detection of molecular markers can be selected as a supplement. Our current results showed that it can be used as a diagnostic molecular marker for PTC.

We analyzed the preoperative general data and experimental data of serum T3, T4, TSH, TGAb, TPOAb and NDRG3 in 81 PTC patients, 75 BTN patients, 77 healthy volunteers during the same period. The results showed that the median of serum NDRG3 was the lowest in PTC group, followed by BTN group, and the highest in HC group. The median value of serum NDRG3 in PTC group was significantly lower than that in the other two groups. Then we did an immunohistochemical experiment and found that the expression of NDRG3 in the tissues of the three groups was consistent with that in blood, which indicated that the production of NDRG3 was reduced or consumed in thyroid papillary carcinoma and thyroid nodules, so a decrease was detected. We speculate that NDRG3 may be involved in the development of this cellular proliferative disease. It has been found that oxygen and lactic acid regulate NDRG3-mediated lactic acid-dependent signaling pathway. Oxygen negatively regulates NDRG3 expression at the protein level through (proline hydroxylase2) PHD2/(von Hippel Lindau disease) VHL system (23). As members of the NDRG family of oxygen sensors, they exhibit tumor suppressive behavior in various cancers, and their expression is considered to be a good prognostic marker (24). In addition, some studies have found that NDRG3 may be a target for controlling the invasive behavior of hypoxic cancer cells (25). But we do not know its performance in oxygen-rich tissues, we know that cellular hypoxia usually occurs in organs with insufficient blood supply or cells with strong metabolism, while the blood supply of thyroid is abundant, so there is little hypoxia. According to the above point of view, under pathological conditions, NDRG3 may increase in anoxic tissues and decrease in oxygen-enriched tissues. However, studies have found that NDRG3 is highly expressed in gastric and prostate cancer (24, 26), while low expression in breast cancer and oral squamous cell carcinoma (21, 27). From the above studies, it seems that the high expression of NDRG3 in anoxic cancer tissues and the low expression in oxygen-rich cancer tissues seems to be unreasonable.

Recent studies have found that the same member of the NDRG family can have both tumor promotion and inhibition at the same time, which may be determined by the tissue specificity of each member. It has been found that NDRG3 is capable of activating RAF-ERK pathway under hypoxia and accelerating tumor occurrence and development through the accumulation of lactic acid (28). NDRG3 regulates meiotic double-strand DNA break repair by regulating ERK signal pathway in male germ cells (29). In addition, NDRG3 also up-regulate the expression of angiogenic chemokine 43 (CXCL1, CXCL3 and CXCL5), enhance the expression of Angiogen-44, thereby ultimately promoting tumor progression (30). However, in our study, the expression of NDRG3 decreased in two diseases with active proliferation of benign and malignant cells. NDRG3 may be involved in the pathogenesis of PTC as a tumor suppressor gene, the reason of which may be related to the metabolic transformation of cancer cells. It has been found that, NDRG3 is degraded under normoxic conditions, but becomes very stable under hypoxic conditions by binding to lactic acid, even in the case of cell reoxygenation (31). Raquel Guimarães Coelho et al. found the Warburg effect in PTC cell line, indicating enhanced glycolysis and lactic acid fermentation in PTC (32). This may prove that the binding of lactic acid and NDRG3 exists in PTC, so NDRG3 is induced to increase, but combined with more, and eventually detected decrease. In addition, the study found that some BTN may have genetic changes, leading to metabolic changes, similar to thyroid cancer (33), so the NDRG3 detectable in BTN is reduced, but there is no more evidence to support this view. Another study found that NDRG3 increase at the early stage of hypoxia and then decrease during persistent hypoxia (34). Therefore, the same type of cancer may have different manifestations in different stages. Our results showed that the expression of NDRG3 was closely related to extrathyroid extension and lymph node metastasis of PTC. The median value of serum NDRG3 in PTC patients with extrathyroid extension and lymph node metastasis was significantly lower than that in PTC patients without both. Our experiments may suggest that the low expression of NDRG3 is related to the malignant degree of PTC. For example, the description of NDRG3 is inconsistent in the study of breast cancer and liver cancer. The expression of NDRG3 is down-regulated in breast cancer patients, especially in the late stage of the disease (21). In another breast cancer study, the expression of NDRG3 protein is increased, which is related to the invasive biological phenotype and poor prognosis of patients with invasive breast cancer (35). The results of Wang showed that the expression of NDRG3 is up-regulated in hepatocellular carcinoma cells, which reversing the malignant phenotype of hepatocellular carcinoma cells (36). The expression of NDRG3 change the sensitivity of cancer cells to chemotherapeutic drugs, which may be a potential therapeutic strategy for the treatment of liver cancer in the future (37). In conclusion, our study found that the low expression of NDRG3 in PTC is associated with poor prognosis.

Therefore, we speculate that the increased expression of NDRG3 in the general population may indicate the occurrence of hypoxic disease or in the early stage of hypoxia. The decrease or increase of NDRG3 in cancer may be related to the state at that time. If the cell hypoxia persists, lactic acid accumulates, NDRG3 is induced, and the binding with lactic acid increases, the detectable amount decreases. On the contrary, if there is no hypoxia in the cells, NDRG3 will not be pathologically induced and can be expressed normally, and relatively more can be detected in the end. In addition, we speculate that the increase of serum NDRG3 in the general population may play a role as tumor suppressor gene, while a role as carcinogenic gene under the combination of NDRG3 and accumulated lactic acid. These contradictory data may be attributed to differences in tumor micro-environment, tumor type or experimental methods. More specimens are essential to be included in the further study, so as to validate our current findings. There remains several limitations in this study. For example, there is a lack of detailed differentiation of PTC variants and studies of NDRG3 in benign counterparts. We only analyzed a small number of cases, which can be call a limited tissue population study, may be biased, and the experimental design needs further research and improvement.

In Conclusion, the low expression of NDRG3 has a certain differential diagnostic value in patients with BTN and PTC. It is related to the occurrence and development of PTC, and may be a potential marker for diagnosing PTC.

## Data availability statement

The original contributions presented in the study are included in the article/supplementary material. Further inquiries can be directed to the corresponding author.

## Ethics statement

The studies involving human participants were reviewed and approved by The Medical Ethics Committee of Gansu Provincial people’s Hospital approved the protocol. The patients/participants provided their written informed consent to participate in this study.

## Author contributions

JHW contributed to the conception and design of the experiment and data analysis. JW, CD and LT un contributed to the acquisition of data. JHW drafted the study, which was revised by JL and JQ. All authors contributed to the article and approved the submitted version.
